# The Role of ^18^F-FDG PET/CT in Multiple Myeloma Staging according to IMPeTUs: Comparison of the Durie–Salmon Plus and Other Staging Systems

**DOI:** 10.1155/2018/4198673

**Published:** 2018-07-30

**Authors:** Shengming Deng, Bin Zhang, Yeye Zhou, Xin Xu, Jihui Li, Shibiao Sang, Wei Zhang

**Affiliations:** Department of Nuclear Medicine, The First Affiliated Hospital of Soochow University, Suzhou, China

## Abstract

We aimed at comparing the Durie–Salmon Plus (DS Plus) staging system based on Italian Myeloma criteria for PET USe (IMPeTUs) with other two staging systems in predicting prognosis of patients with all stages of newly diagnosed multiple myeloma (MM). A total of 33 MM patients were enrolled in this retrospective study. The variation between the DS Plus classification and Durie–Salmon staging system (DSS) or Revised International Staging System (RISS) classification was assessed. When staged by the DSS, patients in stage I and stage II did not reach the median overall survival (OS), and the median OS was 33 months for stage III (*p*=0.3621). When staged by the DS Plus, patients in stage I did not reach the median OS of stage I, and the median OS for stages II and III was 38 and nine months, respectively (*p*=0.0064). When staged by the RISS, patients in stage I did not reach the median OS, and the median OS was 33 and 16 months for stage II and stage III, respectively (*p*=0.0319). The concordances between two staging systems were 0.07 (DS Plus versus DSS) and 0.37 (DS Plus versus RISS), respectively. Multivariate analysis revealed that DS Plus stage III (HR: 11.539, *p*=0.021) and the Deauville score of bone marrow ≥4 (HR: 3.487, *p*=0.031) were independent prognostic factors associated with OS. Both the DS Plus based on IMPeTUs and RISS possessed a better potential in characterizing and stratifying MM patients compared with the DSS. Moreover, DS Plus stage III and the Deauville score of bone marrow ≥4 were reliable prognostic factors in newly diagnosed MM patients.

## 1. Introduction

As a clonal hematologic malignancy, multiple myeloma (MM) is characterized by bone marrow plasma cell infiltration and the presence of serum and urine monoclonal immunoglobulins. The prognosis of MM patients is highly variable; therefore, a reliable staging system is extremely important to optimize appropriate treatment as quickly as possible and avoid irreversible organ damage [1]. The most widely applied staging systems in MM patients include the International Staging System (ISS) and Durie–Salmon staging system (DSS) [2]. However, both of these two staging systems have some limitations. As a powerful and reproducible stage classification, the ISS simply segregates patients into three different groups based on the levels of *β*_2_-microglobulin and serum albumin. However, the ISS-derived outcome can be affected by serum albumin, which is a host factor and not disease-specific [3]. Moreover, the ISS relies solely on tumor biological parameters but does not integrate any medical imaging modalities. The DSS relies on a combination of clinical factors, such as the number of lytic bone lesions on a skeletal radiographic survey, serum calcium, level of hemoglobin, amount of M protein, and renal function [4]. However, the DSS has a poor reproducibility because its classification based on the extent and number of bone lesions found by X-ray is observer-dependent [5].

Recently, the Revised International Staging System (RISS) improves the prognostic value of the ISS by combining the variables in the ISS with the chromosomal abnormalities (CA) detected by interphase fluorescence *in situ* hybridization (t(14;16), t(4;14), and del17p) and serum lactate dehydrogenase (LDH) in those patients with newly diagnosed MM [6]. According to one recent study, the RISS can also be used to stratify patients with relapsed/refractory MM [7].

In 2006, the Durie–Salmon Plus (DS Plus) staging system integrated new imaging techniques, such as ^18^F-2-fluoro-2-deoxyglucose (^18^F-FDG) positron emission tomography/computed tomography (PET/CT) and magnetic resonance imaging (MRI), into a new generation of MM staging and offered the opportunity to precisely stage patients by anatomic and functional techniques [8]. ^18^F-FDG PET/CT provides prognostic information on symptomatic MM at the baseline and therapeutic follow-up, allowing the detection of extramedullary disease (EMD) [9]. However, no standard interpretation criteria have been proposed for the evaluation of ^18^F-FDG PET/CT scans in MM, preventing reproducibility of data.

A group of Italian nuclear medicine experts, medical physicists, and hematologists have defined new visual interpretation criteria (Italian Myeloma criteria for PET USe; IMPeTUs) to standardize the ^18^F-FDG PET/CT interpretation criteria and methods for extensive use in clinical practice for symptomatic MM patients. However, a larger series of patients should be adopted to define a visual cutoff for positivity [10].

It remains unclear whether the DS Plus based on IMPeTUs can be used to appropriately interpret ^18^F-FDG PET/CT imaging in MM patients. Therefore, we aimed at comparing the DS Plus based on IMPeTUs with other staging systems in predicting prognosis of patients with all stages of newly diagnosed MM in this study.

## 2. Materials and Methods

### 2.1. Patients

This retrospective study was approved by the Ethics Committee of the First Affiliated Hospital of Soochow University with waiver of informed consent.

Patients with newly diagnosed monoclonal plasma cell disease who had available data of RISS stage and DSS stage were enrolled in this analysis between May 2007 and December 2017. All patients underwent whole-body ^18^F-FDG PET/CT for initial diagnosis of disease.

Subjects were selected for inclusion if they met the following criteria: (1) the diagnosis of MM confirmed by the presence of an M-component in serum and/or urine plus clonal plasma cells in the bone marrow and/or a documented clonal plasmacytoma, (2) digital image data available for retrospective analysis, and (3) the time interval between assessment of hematological and immunologic parameters and ^18^F-FDG PET/CT <3 weeks.

The following patients were excluded: (1) patients who had insufficient information on the RISS, DSS, and DS Plus, (2) MM patients who had additional diseases, and (3) patients who had received treatment before ^18^F-FDG PET/CT acquisition.

### 2.2. RISS, DSS, and DS Plus

Staging of 33 MM patients was conducted using the RISS, DSS, or DS Plus based on ^18^F-FDG PET/CT imaging and laboratory data (Table 1).

### 2.3. PET/CT Acquisition

All patients underwent whole-body ^18^F-FDG PET/CT according to the standard protocol in the same center. The patients were instructed to fast for at least 6 h. The blood glucose level of all the patients was lower than 11 mmol/L. Next, 60 min after the injection of ^18^F-FDG (dose of 0.12 mCi/kg), imaging was started on a Discovery STE PET/CT scanner (General Electric Medical Systems, Milwaukee, WI, USA) with a CT of the whole body (140 kV, 120 mA, transaxial FOV 700 mm, pitch 1.75, rotation time 0.8 s, and slice thickness 3.75 mm). Subsequently, a whole-body emission scan in a 3D mode was performed from the base of the skull to the midfemur, 2-3 min per bed position. PET images were reconstructed by a standard iterative algorithm (ordered-subset expectation maximization), with the low-dose CT data utilized for attenuation correction and image fusion.

### 2.4. Image Analysis

^18^F-FDG PET images were blindly evaluated by two experienced nuclear medicine physicians in order to avoid any bias on image analysis. Image interpretation of ^18^F-FDG PET/CT was based on IMPeTUs. To make sure IMPeTUs was suitable for DS Plus, bone lesions with a Deauville score ≥4 on PET scan and diffuse lytic lesions ranging between 0.5 and 1 cm in size with a Deauville score ≥3 were all identified as positive bone lesions. For EMD, positive lesions were also defined as the Deauville score ≥4 on PET scan. If there was discordance between the two independent physicians for image analysis, a third investigator was advised to make a decision.

### 2.5. Statistical Analysis

To assess the variation between DS Plus classification and DSS or ISS classification, an exact weighted kappa value was calculated with STATA 11 software package (StataCorp, College Station, TX, USA), which was expressed as a number between 0 and 1, with 0 representing complete nonconcordance and 1 representing complete agreement [11]. To better compare the three staging systems, DS Plus and DSS subgroups were combined within the main numerical grouping for analysis.

OS was determined from the date of the ^18^F-FDG PET/CT scan until the date of death or last follow-up. The OS was estimated by the Kaplan–Meier method. Comparisons among groups were made by a log-rank test. For multivariate analysis, variables that were considered clinically relevant or independently predictive of survival in univariate analysis were introduced into a Cox proportional-hazards model. Statistical analysis was performed by IBM SPSS 19.0 software (SPSS Inc., Chicago, IL, USA) and GraphPad Prism 5.0 software (GraphPad Software Inc., San Diego, CA, USA).

## 3. Results

### 3.1. Characteristics of Patients

A total of 59 MM patients met inclusion criteria for ^18^F-FDG PET/CT. Among them, 20 patients had received treatment before ^18^F-FDG PET/CT acquisition, one patient had myelodysplastic syndromes (MDS), one patient had gastric cancer, one patient had no *β*_2_-MG results, and three patients did not do the examination of cytogenetic abnormalities. According to our exclusion criteria, the abovementioned 26 patients were excluded from the present study. Finally, 33 consecutive patients (10 women, 23 men; mean age ± SD, 60 ± 10 years; range, 34–77 years) were retrospectively enrolled in this study.  Table 2 lists detailed characteristics of patients.

### 3.2. Staging Distribution

**Figure 1:**
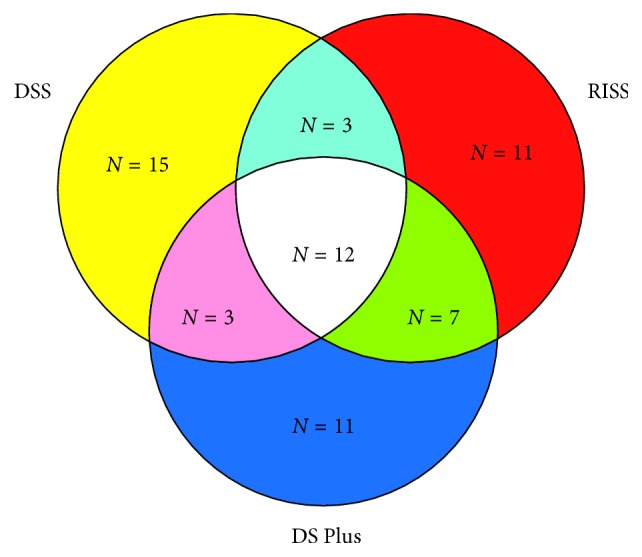
Details of classifications. Different colors represent different staging systems. Red color indicates that when staged by the RISS, the stages of nine patients were different from those staged by the DS Plus and DSS. Blue color indicates that when staged by the DS Plus, the stages of 11 patients were different from those staged by the RISS and DSS. Yellow color indicates that when staged by the DSS, the stages of 15 patients were different from those staged by the RISS and DS Plus. Light blue color indicates that the stages of 3 patients were the same when staged by the DSS and RISS. Green color indicates that the stages of 7 patients were the same when staged by the DS Plus and RISS. Pink color indicates that the stages of 3 patients were the same when staged by the DS Plus and DSS. The total number of patients was 33. Among the 33 MM patients, 12 patients were staged the same according to the three various staging systems (*N* = number of patients).

Table 3 and Figure 1 summarize a detailed classification in MM patients according to the DS Plus staging system compared with RISS or classic DSS classification.

**Figure 2:**
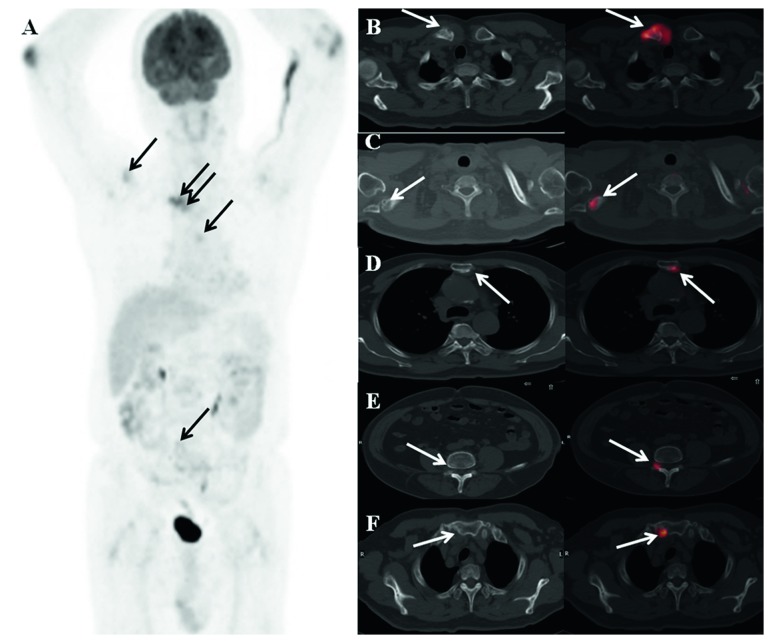
^18^F-FDG PET/CT imaging in a 72-year-old man with MM: (a) MIP image; (b–f) transaxial CT and fused images. In this patient, the descriptive criteria (IMPeTUs) were BM(3), F3.SP.ExtraSP(4), L2, PM, EM.N(2), where BM3 indicates that bone marrow uptake is <liver but >mediastinum, F3 indicates 4 to 10 lesions (arrows), SP indicates the spine, ExtraSP indicates outside the spine with (4) indicating reference lesion uptake >liver uptake + 10%, L2 indicates that 1 to 3 lesions were also lytic, PM indicates a bone lesion in surrounding soft tissues with bone cortical interruption, EM indicates at least one extramedullary lesion, and N indicates that the extramedullary lesion was nodal disease with (2) indicating extramedullary lesion uptake ≤mediastinum. This patient was classified as IIA according to DS Plus.

The comparison of the DS Plus and DSS showed that 45.45% of patients had concordant stages across systems. The patient with DSS stage I MM was upstaged according to the DS Plus staging system. Of two patients with DSS stage II MM, lesions in one patient were staged the same, while lesions in the other patients were downstaged according to the DS Plus staging system. Of 30 patients with DSS stage III MM, lesions in 14 (46.67%) patients were staged the same, while lesions in 16 (53.33%) patients were downstaged according to the DS Plus staging system. Agreement between the DS Plus and DSS stages calculated using the weighted kappa statistic was 0.07 (95% CI, −0.07 to 0.22; *p*=0.167), indicating no concordance between DS Plus and DSS stages. Examples are given in Figure 2.

**Figure 3:**
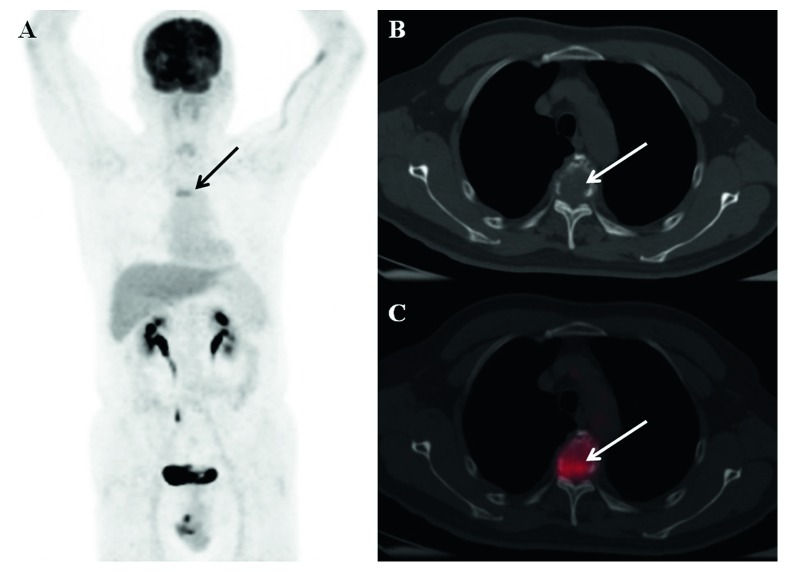
^18^F-FDG PET/CT imaging in a 60-year-old man with MM: (a) MIP image; (b-c) transaxial CT and fused images. In this patient, the descriptive criteria (IMPeTUs) were BM(2), F2.SP(4), L2. ^18^F-FDG PET/CT showed increased ^18^F-FDG uptake in the T4 spine (arrows). His disease stage was IA by DS Plus. The serum *β*_2_-microglobulin level was 6.08 mg/L, and the LDH level was 310 U/L. The patient was classified as III according to the RISS.

The comparison of the DS Plus and RISS showed that 57.58% of patients had concordant stages across systems. Among four patients with RISS stage I MM, one, two, and one were classified as stages I, II, and III based on the DS Plus staging system, respectively. Among 15 patients with RISS stage II MM, six, seven, and two were classified as stages I, II, and III based on the DS Plus staging system, respectively. Among 14 patients with RISS stage III MM, two, one, and 11 were classified as stages I, II, and III based on the DS Plus staging system, respectively. Agreement between the DS Plus and RISS stages calculated using the weighted kappa statistic was 0.37 (95% CI, 0.12–0.62; *p* < 0.01), indicating fair concordance between DS Plus and RISS stages. Examples are given in Figure 3.

### 3.3. Survival according to the Three Staging Systems

**Figure 4:**
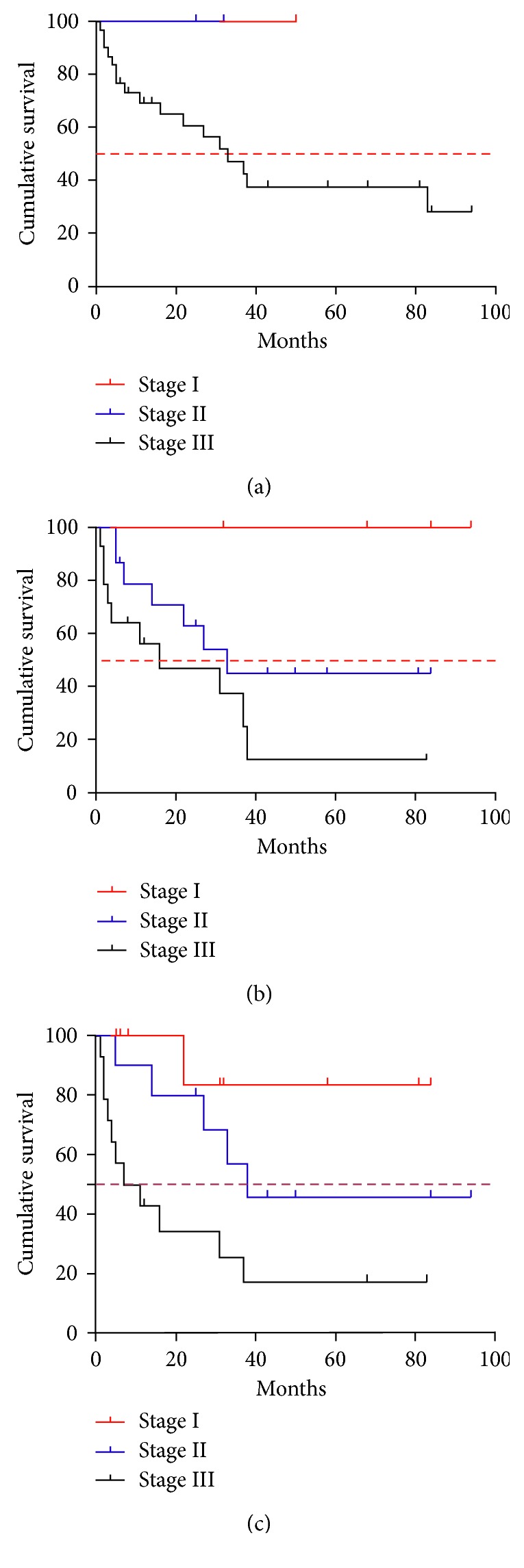
OS of the 33 MM patients according to the DSS (a), RISS (b), and DS Plus (c).

When staged by the DSS, patients in stage I and stage II did not reach the median overall survival (OS), and the median OS was 33 months for stage III (*p*=0.3621; Figure 4(a)). When staged by the RISS, patients in stage I did not reach the median OS of RISS stage I (the median follow-up duration of the stage I patients was 59 months), and the median OS was 33 and 16 months for stage II and stage III, respectively (*p*=0.0319; Figure 4(b)). When staged by the DS Plus, patients in stage I did not reach the median OS of stage I, and the median OS for stages II and III was 38 and nine months, respectively (*p*=0.0064; Figure 4(c)). For the RISS, the 5-year OS rate was 100%, 45.02%, and 12.5% for MM patients in stages I, II, and III, respectively. For the DSS, the 5-year OS rate was 100%, 100%, and 28.29% for MM patients in stages I, II, and III, respectively. For the DS Plus, the 5-year OS rate was 85.71%, 45.71%, and 17.14% for MM patients in stages I, II, and III, respectively.

The *p* value was statistically significant for both DS Plus and RISS, while no statistical significance was noted for the DSS.

### 3.4. Prognostic Factors

According to the univariate analysis, other factors, such as the presence of EMD (*p*=0.015), subgroup B of DS Plus (*p*=0.012), and the Deauville score of bone marrow ≥4 (*p*=0.013), were significantly associated with shorter OS (Table 4).

Multivariate analysis revealed that DS Plus stage III (HR: 11.539, *p*=0.021) and the Deauville score of bone marrow ≥4 (HR: 3.487, *p*=0.031) were independent prognostic factors associated with OS (Table 5).

## 4. Discussion

In recent years, new imaging techniques, such as PET or MRI, play an increasingly important role in staging of MM. The impact of MRI on staging patients according to DS Plus has been analyzed in many studies [12–14]. Although ^18^F-FDG PET/CT has been proved to be prognostically valuable in staging different groups [15–17], only few studies have been reported on DS Plus based on ^18^F-FDG PET/CT [18]. One of the reasons is that no standard interpretation criteria have been proposed for the evaluation of ^18^F-FDG PET/CT scans in MM. Therefore, IMPeTUs has been proposed to standardize image interpretation criteria in order to make clinical trial results applicable and reproducible. Since IMPeTUs is only a descriptive criterion according to experience with ^18^F-FDG PET/CT in lymphoma and MM [17, 19, 20], we decided to adopt the Deauville score ≥4 and diffuse lytic lesions with the Deauville score ≥3 as the cutoff values in this study. Our study confirmed that IMPeTUs was useful to define bone lesions and EMD, which were main criteria of the DS Plus. DS Plus based on IMPeTUs showed fair concordance with the classic RISS, indicating great capability to differentiate patients with good OS from those with a poorer prognosis.

To the best of our knowledge, the present study was the first study designed to compare the DSS, RISS, and DS Plus. In some studies, researchers have compared the DSS with the ISS and found that the ISS has a better reliability, simplicity, and predictability for OS compared with the DSS for MM patients [5, 21, 22]. The RISS, which combines the ISS together with the prognostic power of high-risk CA and LDH, has been proved to give a better differentiation of MM patients into three survival subgroups [6, 7, 23, 24]. Our data also indicated that the RISS provided significant prognostic information.

Nowadays, it remains largely explored whether the DSS can be used to predict the outcome. Most studies have demonstrated that the DSS cannot show a significant difference in OS among stages I, II, and III [21, 22, 25], while several studies have reported that there are statistical differences in OS among the three groups in the DSS [2, 26]. In the present study, we found that the DSS was not correlated with OS. In most of the studies comparing the frequency distribution of the same patients across the DSS and ISS, the number of patients classified as DSS stage III is larger than that of patients classified as ISS stage III [5, 21, 22, 25, 26]. The percentage of patients with DSS stage III in our study was also higher. The reason might be attributed to that the DSS includes hemoglobin and serum calcium, which can be affected by various factors and advanced lytic bone lesions in X-ray, leading to difficulty in appropriate interpretation due to the lack of standard criteria.

It is believed that DS Plus is a reliable method for both staging and prognostic classification. The prognostic significance of DS Plus has been reported in one recent study using MRI of the spine and pelvis in 85 patients [27]. However, Fechtner et al. have reported that the DS Plus is not better than the DSS for the prediction of OS [28]. Although only few studies have reported the DS Plus in combination with ^18^F-FDG PET/CT, the prognostic implication of PET/CT has been documented in several articles [16, 29, 30]. Our study confirmed that DS Plus based on ^18^F-FDG PET/CT has a great potential in predicting OS of MM patients.

DS Plus classification is mainly based on the number of focal bone lesions and the serum creatinine level and/or the presence of EMD. ^18^F-FDG PET/CT has a superior detection rate of focal bone lesions compared with whole-body X-ray and MRI of the spine and pelvis. Zamagni et al. have shown that ^18^F-FDG PET/CT has a better sensitivity than whole-body X-ray in 46% of patients, and it enables the detection of myelomatous lesions at sites that are not within the field of view (FOV) of MRI in 35% of patients [31]. Fonti et al. have prospectively evaluated 33 newly diagnosed MM patients by comparing ^18^F-FDG PET/CT with MRI of the spine and pelvis. They have found that MRI is better in the detection of diffuse diseases, while ^18^F-FDG PET/CT detects a considerable number of focal bone lesions that are out of the FOV of MRI [29]. The prognostic value of the number of focal bone lesions on ^18^F-FDG PET/CT has been confirmed in several studies [9, 16]. DS Plus could discriminate patients of different stages in this study, which might be attributed to accurate assessment of the number and site of focal PET positive bone lesions with or without osteolytic characteristics detected by ^18^F-FDG PET/CT.

In addition, criteria of IMPeTUs include the description of the metabolic state of the bone marrow. In a study of 18 MM patients, clinical course of the disease could be predicted by the associated ^18^F-FDG bone marrow images [32]. However, in other studies, ^18^F-FDG uptake of the bone marrow in PET/CT is not significantly associated with OS [29, 33]. The reason might be attributed to that the intensity of accumulation was assessed for ^18^F-FDG images using different methods. In our study, the univariate and multivariate analyses showed that the metabolic state of the bone marrow was an independent prognostic factor associated with OS.

There were some limitations in the present study: First, the sample size was small. Stage I patients in the DS Plus, RISS, and DSS all survived due to limited patient number. Because PET/CT is not yet a standardized imaging tool in MM, only a few MM patients performed PET/CT based on physicians' advice. In addition, some patients were excluded because of incomplete data. Second, in the present study, positive bone lesions were defined as lesions with a Deauville score ≥4 on PET scan and diffuse lytic lesions ranging between 0.5 and 1 cm in size with a Deauville score ≥3, and such a definition might reduce the specificity and consequently increase the false-positive rate. However, lesions in 51.5% of patients were downstaged according to the DS Plus compared with the DSS in our study. Therefore, we think that this definition is useful to guide therapy. However, a prospective study with a larger population should be adopted to define a cutoff for positivity.

Taken together, our findings indicated that DS Plus based on IMPeTUs was useful for the initial staging of MM. Both DS Plus and RISS possessed a better potential in predicting OS of MM patients compared with the DSS. DS Plus stage III and the Deauville score of bone marrow ≥4 were independent prognostic factors associated with OS.

## Figures and Tables

**Table 1:** Criteria of RISS, DSS, and DS Plus staging systems.

	RISS [6]	DSS [4]	DS Plus [8]
Stage I	ISS stage I (serum albumin ≥3.5 and serum *β*_2_-microglobulin <3.5), normal LDH levels, and no high-risk cytogenetic abnormalities	All of the following: hemoglobin value >10 g/dL; serum calcium value normal or ≤10.5 mg/dL; bone X-ray shows normal bone structure or solitary bone plasmacytoma only; and low M-component production rates (IgG < 5 g/dL, IgA < 3 g/dL, and Bence Jones protein < 4 g/24 h)	Stage IA, smoldering or indolent: single plasmacytoma and/or limited disease at imaging Stage IB: 0–4 focal lesions or mild diffuse disease

Stage II	Neither stage I nor stage III	Neither stage I nor stage III	5–20 focal lesions or moderate diffuse disease

Stage III	ISS stage III (serum *β*_2_-microglobulin >5.5 mg/L) and either elevated LDH levels or high-risk cytogenetic abnormalities	One or more of the following: hemoglobin value <8.5 g/dL; serum calcium value <12 mg/dL; advanced lytic bone lesions; and high M-component production rates (IgG > 7 g/dL, IgA > 5 g/dL, or Bence Jones protein >12 g/24 h)	>20 focal lesions or severe diffuse disease

Subgroup A	—	Relatively normal renal function: serum creatinine value <2.0 mg/dL	Serum creatinine level <2.0 mg/dL and no EMD

Subgroup B	—	Abnormal renal function: serum creatinine value ≥2.0 mg/dL	Serum creatinine level >2.0 mg/dL and/or the presence of EMD

**Table 2:** Characteristics of patients.

Number	Sex	Age	Myeloma type	IMPeTUs	DSS	DS Plus	RISS	Treatment
1	M	57	IgG *λ*	BM(4)A, F4.S.SP.ExtraSP(5), L4, PM, EM.EN(4)	B	IIIB	III	None
2	M	38	IgG *κ*	BM(3), F4.SP.ExtraSP(3), L4, EM.N.EN(4)	IIIA	IIIB	III	Chemotherapy
3	M	63	Nonsecretory	BM(2), F4.SP.ExtraSP(4), L4, PM, EM.EN(3)	IIIB	IIB	III	None
4	M	68	Light chain *κ*	BM(2), F4.SP.ExtraSP(4), L4, PM, EM.EN(3)	IIIB	IIIB	III	Chemotherapy
5	F	48	IgG *κ*	BM(2), F4.SP.ExtraSP(4), L4, PM	IIIA	IIA	I	Chemotherapy + IMIDs
6	M	55	IgG *κ*	BM(2), F4.SP.ExtraSP(4), L1, PM	IIIA	IIA	I	Chemotherapy + IMIDs
7	M	58	Light chain *λ*	BM(2), F3.SP.ExtraSP(4), L3, PM	IIIA	IA	II	Chemotherapy
8	M	63	IgG *λ*	BM(2), F4.S.SP.ExtraSP(4), L4	IIIA	IIIA	I	Chemotherapy
9	M	58	IgG *κ*	BM(3), F4.SP.ExtraSP(4), L4, PM, EM.N.EN(5)	IIIB	IIIB	III	Chemotherapy
10	F	42	IgG *κ*	BM(4), F1, L1	IIIA	IA	II	Chemotherapy + ASCT
11	M	60	Light chain *κ*	BM(3), F4.SP.ExtraSP(4), L4, EM.EN(3)	IIIA	IIA	II	Chemotherapy + ASCT
12	M	63	IgG *κ*	BM(2), F2.ExtraSP(4), L1	IIIA	IA	II	Chemotherapy
13	M	52	IgG *λ*	BM(3)A, F4.S.SP.ExtraSP(3), L2, EM.EN(3)	IIIA	IIIA	III	None
14	M	67	IgG *λ*	BM(4), F3.ExtraSP(4), L2	IIIA	IIA	II	Chemotherapy + allo-BMT
15	F	71	Light chain *κ*	BM(5), F4.SP.ExtraSP(5), L3, PM, EM.N(5)	IIIA	IIIB	II	Chemotherapy + radiotherapy
16	F	64	IgG *κ*	BM(3), F4.S.SP.ExtraSP(3), L3	IIIB	IIIB	III	Chemotherapy + IMIDs
17	F	67	IgG *κ*	BM(4)A, F4.SP.ExtraSP(4), L4	IIIB	IIIB	III	None
18	M	34	IgG *κ*	BM(3), F4.SP.ExtraSP(5), L3, PM, EM.EN(4)	IIIA	IIB	II	None
19	M	77	IgA *κ*	BM(2)A, F3.SP(4), L1, PM, EM.N.EN(3)	IIIA	IIA	II	None
20	M	72	IgG *κ*	BM(3), F3.SP.ExtraSP(4), L2, PM, EM.N(2)	IA	IIA	II	Chemotherapy
21	M	60	IgG *λ*	BM(3), F4.SP.ExtraSP(5), L3, PM, EM.N(5)	IIIA	IIB	II	Chemotherapy + IMIDs
22	M	73	Light chain *κ*	BM(3), F4.SP.ExtraSP(5), L2, EM.EN(5)	IIIB	IIIB	III	Chemotherapy + IMIDs
23	M	60	IgD *λ*	BM(2), F2.SP(4), L2	IIIA	IA	III	Chemotherapy + IMIDs
24	M	59	Light chain *κ*	BM(3), F3.SP.ExtraSP(5), L1, PM, EM.N(5)	IIA	IB	I	None
25	M	64	IgG *κ*	BM(2), F2.SP(4), L4, PM	IIB	IIB	II	Chemotherapy + IMIDs
26	F	57	IgG *λ*	BM(4)A, F4.SP.ExtraSP(4), L4, PM, EM.N(5)EN(4)	IIIA	IIIB	II	Chemotherapy
27	F	76	Light chain *κ*	BM(4)A, F4.SP.ExtraSP(5), L4, PM, EM.EN(5)	IIIA	IIIB	III	None
28	F	58	IgA *λ*	BM(4)A, F4.S.SP.ExtraSP(5), L4, EM.EN(5)	IIIA	IIIB	III	Chemotherapy + IMIDs
29	F	47	Light chain *κ*	BM(2), F2.SP(5), L2, PM, EM.EN(5)	IIIA	IB	II	Chemotherapy + radiotherapy
30	M	69	IgG *λ*	BM(2), F2.SP(2), L2, EM.EN(4)	IIIB	IB	II	Chemotherapy
31	M	65	IgG *λ*	BM(2), PM, EM.EN(4)	IIIA	IB	III	Chemotherapy + ASCT
32	F	53	IgD *λ*	BM(4)A, EM.EN(4)	IIIB	IB	II	Chemotherapy + IMIDs
33	M	50	IgG *λ*	BM(5)A, F4.S.SP.ExtraSP(5), L4, EM.EN(4)	IIIB	IIIB	III	Chemotherapy

ASCT: autologous stem cell transplant; IMIDs: immunomodulatory drugs; BM: bone marrow; F: focal bone lesions; S: skull; Sp: spine; L: lytic lesion; Fr: fracture; EM: EMD; PM: paramedullary disease; N: nodal disease; EN: extranodal disease.

**Table 3:** Correlation of classification according to DS Plus, DSS, and RISS.

DS Plus	DSS			Total	RISS			Total
	Stage I	Stage II	Stage III		Stage I	Stage II	Stage III	
Stage I	0	1	8	9	1	6	2	9
Stage II	1	1	8	10	2	7	1	10
Stage III	0	0	14	14	1	2	11	14
Total	1	2	30	33	4	15	14	33

**Table 4:** Results of univariate analysis of the factors that influence the OS.

Characteristics	*n*=33 (%)	Median OS (months)	*p* value
*Sex*			
Male	23 (69.7)	37	0.721
Female	10 (30.3)	22	

*Age*			
<60	15 (45.5)	31	0.915
≥60	18 (54.5)	33	

*Type*			
IgG	21 (63.6)	NR	0.243
IgA	1 (3.0)	33	
Light chain	8 (24.2)	7	
Others	3 (9.1)	38	

*Bone marrow*			
Deauville score ≥4	10 (30.3)	7	0.013
Deauville score <4	23 (69.7)	38	

*Paramedullary disease*			
+	17 (51.5)	33	0.513
−	16 (48.5)	37	

*Extramedullary disease*			
+	16 (48.5)	16	0.015
−	17 (51.5)	NR	
*Creatinine*			
<2 mg/dl	22 (66.7)	37	0.064
≥2 mg/dl	11 (33.3)	16	

*Subgroup of DS Plus*			
A	12 (36.4)	NR	0.012
B	21 (63.6)	16	

*Treatment*			
None	11 (33.3)	31	0.146
Chemotherapy	7 (21.2)	37	
Chemotherapy + IMIDs	9 (27.3)	NR	
Chemotherapy + BMT	4 (12.1)	27	
Others	2 (6.1)	7	

NR: not reached; BMT: bone marrow transplant.

**Table 5:** Multivariate analysis of factors associated with OS in univariate analysis, with DS Plus in the model.

	HR	95% CI	*p* value
DS Plus-I (reference)	1	—	—
DS Plus-II	4.818	0.547–42.40	0.156
DS Plus-III	11.539	1.453–91.638	0.021
Deauville score of BM ≥4	3.487	1.121–10.850	0.031
Presence of EMD	1.463	0.350–6.107	0.602
DS Plus subgroup B	2.014	0.416–9.762	0.384
